# Gut Microbiota and Phytoestrogen-Associated Infertility in Southern White Rhinoceros

**DOI:** 10.1128/mBio.00311-19

**Published:** 2019-04-09

**Authors:** Candace L. Williams, Alexis R. Ybarra, Ashley N. Meredith, Barbara S. Durrant, Christopher W. Tubbs

**Affiliations:** a Institute for Conservation Research, San Diego Zoo Global, Escondido, California, USA; b Institute for Genomics, Biocomputing and Biotechnology, Mississippi State University, Mississippi State, Mississippi, USA; c Mississippi State Chemical Laboratory, Mississippi State, Mississippi, USA; University of Utah; University of Hawaii at Manoa

**Keywords:** endocrine disruption, fertility, gut microbiomes, phytoestrogens, rhinoceros

## Abstract

With recent poaching of southern white rhinoceros (SWR [*Ceratotherium simum simum*]) reaching record levels, the need for a robust assurance population is urgent. However, the global captive SWR population is not currently self-sustaining due to the reproductive failure of captive-born females. Dietary phytoestrogens have been proposed to play a role in this phenomenon, and recent work has demonstrated a negative relationship between diet estrogenicity and fertility of captive-born female SWR. To further examine this relationship, we compared gut microbial communities, fecal phytoestrogens, and fertility of SWR to those of another rhinoceros species—the greater one-horned rhinoceros (GOHR [*Rhinoceros unicornis*]), which consumes a similar diet but exhibits high levels of fertility in captivity. Using 16S rRNA amplicon sequencing and mass spectrometry, we identified a species-specific fecal microbiota and three dominant fecal phytoestrogen profiles. These profiles exhibited various levels of estrogenicity when tested in an *in vitro* estrogen receptor activation assay for both rhinoceros species, with profiles dominated by the microbial metabolite equol stimulating the highest levels of receptor activation. Finally, we found that SWR fertility varies significantly not only with respect to phytoestrogen profile, but also with respect to the abundance of several bacterial taxa and microbially derived phytoestrogen metabolites. Taken together, these data suggest that in addition to species differences in estrogen receptor sensitivity to phytoestrogens, reproductive outcomes may be driven by the gut microbiota’s transformation of dietary phytoestrogens in captive SWR females.

## Abstract (precis)

Southern white rhinoceros (SWR) poaching has reached record levels, and captive infertility has rendered SWR assurance populations no longer self-sustaining. Previous work has identified dietary phytoestrogens as a likely cause of this problem. Here, we investigate the role of gut microbiota in this phenomenon by comparing two rhinoceros species to provide the first characterizations of gut microbiomes for any rhinoceros species. To our knowledge, our approach, combining parallel sequencing, mass spectrometry, and estrogen receptor activation assays, provides insight into the relationship between microbially mediated phytoestrogen metabolism and fertility that is novel for any vertebrate species. With this information, we plan to direct future work aimed at developing strategies to improve captive reproduction in the hope of alleviating their threat of extinction.

## INTRODUCTION

The southern white rhinoceros (SWR [*Ceratotherium simum simum*]) has returned from the brink of extinction through extensive *in situ* and *ex situ* conservation efforts, with wild populations increasing from approximately 100 to 20,000 over the last century (1). However, wild SWR now face an uncertain future due to the recent dramatic increase in poaching (2). An additional challenge facing the species is the reproductive failure of the once robust *ex situ* assurance populations (3, 4). Together, poaching, long gestational length (∼16 months) and intercalving interval (∼2.5 years) (5), and captive infertility (3, 4) have rendered both wild and captive populations no longer self-sustaining. Without any change in poaching rates, wild SWR populations will likely face the threat of extinction within the next 2 decades (6).

Previous work has implicated captive diets in the reproductive failure of captive SWR (4, 7). In the wild, SWR are pure grazers, consuming up to ∼40 kg/day of various grasses (8, 9). In contrast, diets in managed settings typically contain phytoestrogen-rich legume hays and soy- and alfalfa-based concentrated feeds (4). A survey of nine SWR-breeding institutions demonstrated that diet estrogenicity was strongly associated with the amount of soy- and/or alfalfa-based pellets fed. Moreover, female SWR born at institutions feeding highly estrogenic diets exhibit lower fertility than female SWR born at institutions feeding low-phytoestrogen diets (4).

Due to their structural similarity to endogenous estrogens, phytoestrogens may interact with estrogen receptors (ERs) and disrupt normal endocrine function, reproduction, and development (10–14). Previously, we showed that SWR ERs exhibit higher maximal activation by phytoestrogens than ERs of the greater one-horned rhinoceros (GOHR [*Rhinoceros unicornis*]) (4). Both species consume similar high-phytoestrogen diets in captivity, but GOHR do not exhibit the decrease in fertility observed in SWRs. These data suggest that at the receptor level, SWR are particularly vulnerable to the deleterious effects of phytoestrogen exposure. Whether SWR possess additional species-specific characteristics that predispose them to phytoestrogen sensitivity remains unclear.

Due to the limitations of collecting biological samples from a threatened megafaunal species, little is understood about the specific physiological consequences of SWR consuming estrogenic diets. Altered endocrine and reproductive function by phytoestrogen exposure has been described in humans, rodents, and livestock species (11–14). Many of these effects, including reproductive tract pathologies, erratic or absent luteal activity, and reduced fertility, parallel findings in captive female SWR (15–17). However, the potential role of phytoestrogens in the onset of these pathologies has not been investigated. In other species, the physiological outcomes of phytoestrogen exposure are profoundly affected by transformation of parent compounds following consumption. For example, in ewes, reproductive pathologies and infertility develop following consumption of diets high in the isoflavone daidzein (DZ), but it is equol (EQ), a daidzein metabolite, that is thought to be the driver of this effect (11). Equol production relies exclusively on microbial transformation, and several other phytoestrogens are metabolized by members of the gastrointestinal tract microbiota to produce metabolites that vary in estrogenicity (18–21). Coumestrol (CO), a compound from another class of phytoestrogens, the coumestans, also has been associated with sheep infertility (12), but to date, the microbial metabolism of coumestans has not been explored. Whether gut microbiota may play a similar role in SWR responses to dietary phytoestrogens is unclear.

The relationship between animals and their associated microbes is important, as microbiota are essential for many biological processes within their hosts (22). However, an understanding of how interactions between phytoestrogens and resident gut microbiota may affect fertility is lacking for any vertebrate species. Given what is known about bioactivation of phytoestrogens by gut microbiota in other mammalian species (23) and the strong link between dietary phytoestrogens and reproductive failures in rhinoceros (4), an investigation into phytoestrogen metabolism by rhinoceros gut microbiota is warranted. To examine these interactions, we characterized SWR and GOHR fecal microbiota as a proxy for gut microbiota. In addition, we compared fecal phytoestrogen composition and metabolite profile estrogenicity, using mass spectrometry (MS) and ER activation assays, respectively, between the two species. By sampling separately housed but similarly managed SWR and GOHR females from the same institution, we sought to reduce variation by eliminating known drivers of gut microbiota composition, such as diet and geographic location (24–26), to better identify species differences. Finally, we used historical breeding records to examine the relationships between specific microbial taxa, phytoestrogen metabolites, and SWR reproductive success. With these data, we shed light on the role microbiota may play in captive SWR infertility with the aim to develop techniques to support and increase this species’ assurance population.

## RESULTS

### Composition of fecal microbiota, but not phytoestrogens, differs by species.

**FIG 1:**
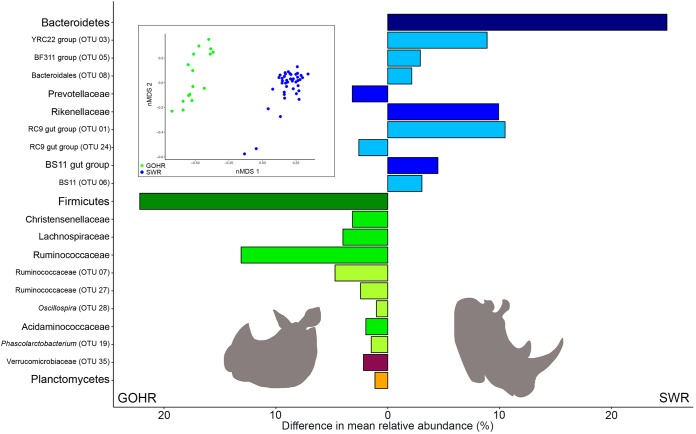
Differences in fecal microbiota between southern white rhinoceros (SWR) and greater one-horned rhinoceros (GOHR). Shown is nonmetric multidimensional scaling (nMDS [inset]) analysis displaying differences in microbiota observed by 16S rRNA amplicon sequencing based on Bray-Curtis distances (PERMANOVA, *P* < 0.001; stress, 0.13). Differences in mean relative abundance of bacterial taxa found to significantly contribute to variation between rhinoceros species (SIMPER, ≥2.0%; Welch’s *t* test, *P* < 0.05) are organized by color, with all members of a particular phylum sharing a similar color, with intensity decreasing from phylum to family to OTU level.

**FIG 2:**
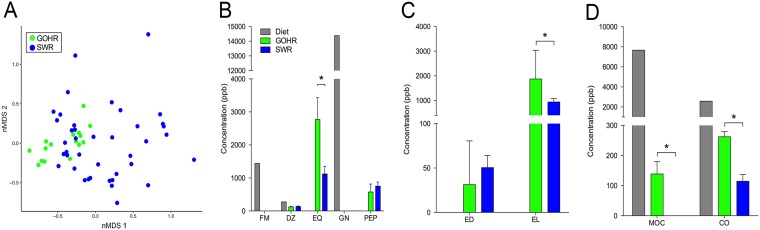
Comparison of fecal phytoestrogen compositions between southern white rhinoceros (SWR) and greater one-horned rhinoceros (GOHR). (A) Nonmetric multidimensional scaling (nMDS) analysis displaying overall composition of fecal phytoestrogens detected by mass spectrometry based on Bray-Curtis distances (PERMANOVA, *P* > 0.05; stress, 0.13). (B to D) Mean ± SE analyte concentrations in parts per billion (ppb) of (B) isoflavones, (C) lignans, and (D) coumestans for both SWR and GOHR and their diet. *, significantly different concentrations of fecal analytes (Welch’s *t* test, *P* < 0.05). FM, formononetin; DZ, daidzein; EQ, equol; GN, genistein; PEP, 4′-ethylphenol; ED, enterodiol; EL, enterolactone; MOC, methoxycoumestrol; CO, coumestrol.

Sequencing of 16S rRNA from fecal samples (SWR, *n *=* *42; GOHR, *n *=* *16 [see Table S1 in the supplemental material]) collected from eight individual rhinoceros (SWR, *n *=* *6; GOHR, *n *=* *2 [Table S1]) revealed that GOHR samples had significantly higher intersample diversity compared to SWR, despite SWR having a higher number of unique, low (<1%)-relative-abundance operational taxonomic units (OTUs) overall (see Table S2 in the supplemental material). Significant differences in fecal community structure and composition between rhino species were also observed at the phylum, family, and OTU levels using permutational analysis of variance (PERMANOVA) and accounting for relative abundances using weighted UniFrac (all *P* < 0.001). A difference in microbial communities was also observed by nonmetric multidimensional scaling (nMDS) (Fig. 1, inset). Members of four phyla were found to significantly contribute to variation (Fig. 1), with the relative abundance of the *Bacteroidetes* (SWR, 55% ± 1.1%; GOHR, 30% ± 1.8%) and the *Firmicutes* (SWR, 33% ± 1.2%; GOHR, 55% ± 2.2%) differing significantly with respect to rhino species (Welch’s *t* test, both *P* < 0.001) (Fig. 1). Several members of these phyla were also found to be significantly different at both the family and OTU levels, with 6 families and 11 OTUs contributing to these significant differences (Fig. 1). Despite species differences in microbial communities, neither overall structure nor composition of detected phytoestrogen analytes varied significantly between SWR and GOHR (PERMANOVA, *P* > 0.05) (Fig. 2A). However, species differences were observed at the individual analyte level. Concentrations of equol (EQ), enterolactone (EL), methoxycoumestrol (MOC), and coumestrol (CO) were significantly higher in the GOHR (Fig. 2B to D; see Table S3 in the supplemental material). Several phytoestrogens were detected exclusively in the diet. These included the isoflavones, formononetin (FM) and genistein (GN) (Fig. 2B), whereas microbially derived metabolites EQ, 4’-ethylphenol (PEP), EL, and enterodiol (ED) were detected only in feces (Fig. 2B and C). Two other phytoestrogens, biochanin-A and *o*-demethylangolesin, were not detected in any sample type (both, <65 ppb). In general, there was an overall trend for excreted quantities of phytoestrogens and metabolites to be higher in GOHR compared to SWR (Fig. 2B to D).

10.1128/mBio.00311-19.3 TABLE S1 Study animals. Download Table S1, DOCX file, 0.1 MB. Copyright © 2019 Williams et al. 2019 Williams et al. This content is distributed under the terms of the Creative Commons Attribution 4.0 International license.

10.1128/mBio.00311-19.4 TABLE S2 Number of sequences, estimate coverage, diversity, and OTU richness of samples. Download Table S2, DOCX file, 0.1 MB. Copyright © 2019 Williams et al. 2019 Williams et al. This content is distributed under the terms of the Creative Commons Attribution 4.0 International license.

10.1128/mBio.00311-19.5 TABLE S3 Significant contributions to variation to species differences and phytoestrogen profiles by individual analytes. Download Table S3, DOCX file, 0.1 MB. Copyright © 2019 Williams et al. 2019 Williams et al. This content is distributed under the terms of the Creative Commons Attribution 4.0 International license.

**FIG 3:**
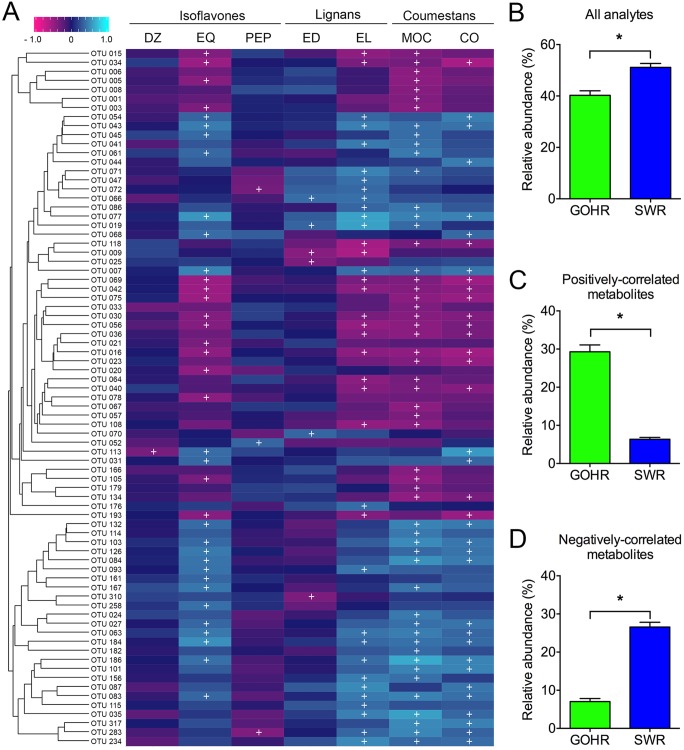
Relative abundance of OTUs and phytoestrogen concentrations significantly correlate. (A) Heat map depicting significant correlations between phytoestrogen analytes and microbiota (≥1.0% relative abundance) using the Spearman correlation method with FDR correction (+ indicates significance at *P* < 0.05). The dendrogram displays OTUs that commonly co-occur by hierarchical clustering (Bray-Curtis), with taxonomic information found in Table S7. (B to D) Species differences in mean ± SE relative abundance of observed OTUs correlating to (B) phytoestrogen analytes, (C) positively correlated metabolites, and (D) negatively correlated metabolites. *, *P* < 0.05 by Welch’s *t* test. DZ, daidzein; EQ, equol; PEP, 4′-ethylphenol; ED, enterodiol; EL, enterolactone; MOC, methoxycoumestrol; CO, coumestrol; GOHR, greater one-horned rhinoceros; SWR, southern white rhinoceros.

The relative abundances of specific OTUs provide some insight into the observed phytoestrogen and metabolite concentrations described above. Overall, 77 OTUs were found to significantly correlate with the concentration of at least one of the phytoestrogens examined (Fig. 3A), which were overall significantly more abundant in SWR compared to GOHR (Welch’s *t* test, *P* < 0.0001) (Fig. 3B). Eleven of these OTUs correlated to parent compounds only. Of the 66 OTUs that had significant correlations to metabolites, only two OTUs had both positive and negative interactions with different metabolites: an unclassified *Lachnospiraceae* (OTU 72) with a positive correlation to EL and negative one to PEP and a member of the RFP12 group (OTU 283) with positive correlations to EL and CO and a negative correlation to PEP. When examining metabolites in particular, the 27 OTUs that were negatively correlated with concentration are nearly four times more abundant in SWR, while the 41 positively correlated OTUs are approximately 5-fold less abundant in SWR (both Welch’s *t* test*, P* < 0.0001) (Fig. 3C and D). Taken together, these findings provide a plausible explanation for why there was an overall trend of lower concentrations of individual phytoestrogen analytes in SWR samples.

10.1128/mBio.00311-19.9 TABLE S7 OTUs. Download Table S7, CSV file, 0.1 MB. Copyright © 2019 Williams et al. 2019 Williams et al. This content is distributed under the terms of the Creative Commons Attribution 4.0 International license.

### Three distinct phytoestrogen profiles examined.

**FIG 4:**
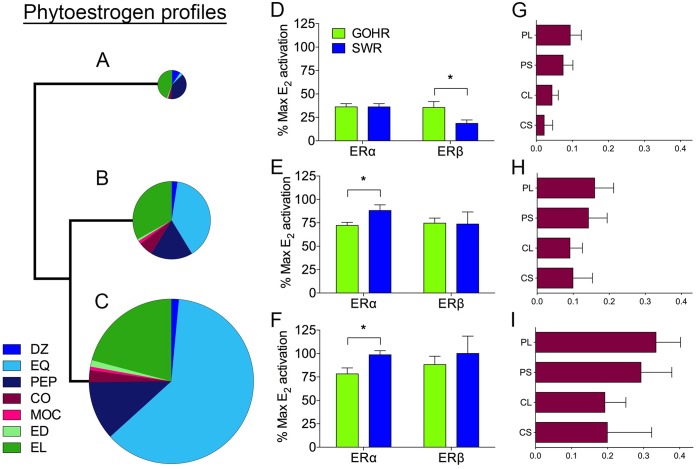
Relative estrogenicity and fertility of phytoestrogen profiles identified by hierarchical clustering. (A to C) Phytoestrogen composition, as depicted by hierarchical clustering, with each profile’s size relative to total concentration detected by mass spectrometry for (A) profile A, (B) profile B, and (C) profile C. (D to F) Mean ± SE activation of ERα and ERβ of both southern white rhinoceros (SWR) and greater one-horned rhinoceros (GOHR) relative to maximal activation by 17β-E_2_ by the respective phytoestrogen profiles for (D) profile A, (E) profile B, and (F) profile C, when tested at concentrations found *in vivo*. (G to I) Differences in mean ± SE fertility measurements with respect to phytoestrogen profiles for (G) profile A, (H) profile B, and (I) profile C. *, significantly different activation (ANOVA, *P* < 0.05). DZ, daidzein; EQ, equol; PEP, 4′-ethylphenol; ED, enterodiol; EL, enterolactone; MOC, methoxycoumestrol; CO, coumestrol; PL, Pregnancy_Life_; PS, Pregnancy_Study_; CL, Calf_Life_; CS, Calf_Study_.

With no clear species difference in metabolite composition, hierarchical clustering was used to group similar fecal samples from both species of rhinoceros according to their phytoestrogen composition. This approach identified three distinct phytoestrogen profiles representing the most commonly observed fecal metabolite profiles in individual samples from both SWR and GOHR (Fig. 4A to C; see Fig. S1D in the supplemental material). For the two most similar profiles, the moderately estrogenic EQ was the dominant metabolite produced, followed by the weakly estrogenic EL (profiles B and C) (Fig. 4B and C, Table S3, and Fig. S1). However, total phytoestrogen concentrations in profile C were approximately twice the total concentration of phytoestrogens detected profile B (8,884 ± 970 ppb and 4,254 ± 315 ppb, respectively) (Fig. 4B and C and Fig. S1). A third, less similar profile was also identified, in which the dominant metabolite was EL (profile A) (Fig. 4A and Table S3). The total concentration of phytoestrogens in this profile was significantly lower (1,510 ± 229 ppb) (Fig. 4A and Fig. S1). Despite there being no visual difference in the overall communities using nMDS (Fig. S1E), several bacterial taxa were found to differ significantly with respect to phytoestrogen profiles (a member of YRC22 [OTU 03] and two unclassified *Ruminococcaceae* [OTU 07 and OTU 27]). However, no individual OTU contributed to variation of >8.5%, indicating that a group of microbiota, not individual OTUs, may be important in driving differences between phytoestrogen profiles.

10.1128/mBio.00311-19.1 FIG S1 Differences in phytoestrogen profiles as they relate to individual analyte concentrations, overall microbiota and metabolite composition, and activation of estrogen receptors. (A to C) Mean ± SE fecal concentrations of (A) isoflavones, (B) lignans, and (C) coumestans for the three phytoestrogen profiles determined by hierarchical clustering. (D and E) Nonmetric multidimensional scaling (nMDS) analysis displaying differences in southern white rhinoceros (SWR) fertility overlaying (D) detected fecal phytoestrogens (PERMANOVA, *P* < 0.001; stress, 0.13) and (E) microbiota as observed by 16S rRNA amplicon weighted UniFrac distances (PERMANOVA, *P* = 0.031; stress, 0.13) for the three phytoestrogen profiles (A, B, and C) determined by hierarchical clustering. CS, calf-based/study period; PS, pregnancy-based/study period; CL, calf-based/lifetime; PL, pregnancy-based/lifetime. (F to H) Mean ± SE activation of ERα and ERβ of both southern white rhinoceros (SWR) and greater one-horned rhinoceros (GOHR) relative to maximal activation by 17β-E_2_ by respective phytoestrogen profiles for (F) profile A, (G) profile B, and (H) profile C. Download FIG S1, TIF file, 0.5 MB. Copyright © 2019 Williams et al. 2019 Williams et al. This content is distributed under the terms of the Creative Commons Attribution 4.0 International license.

To quantify the relative estrogenicity of the three dominant phytoestrogen profiles found in fecal samples (profiles A, B, and C), each observed mixture was formulated *in vitro* and tested in estrogen receptor (ER) activation assays using ERα or ERβ from SWR and GOHR (Fig. 4D to F and Fig. S1F and G), as described previously (5). All three phytoestrogen profiles activated SWR and GOHR ERs (Fig. 4D to F and Fig. S1F and G), with profile C the most potent agonist for both SWR ERs reaching maximal activation relative to 17β-estradiol (E_2_) (Fig. 4F; see Fig. S1G and Table S4 in the supplemental material). Similarly, profile B stimulated maximal activation of SWR ERα and near-maximal activation of SWR ERβ relative to E_2_, despite having less than half the total concentration of analytes of profile C (Fig. 4E, Fig. S1F and G, and Table S4). In contrast, the least potent profile, A, stimulated significantly greater activation of GOHR ERβ relative to SWR ERβ (Fig. 4D, Fig. S1F and G, and Table S4).

10.1128/mBio.00311-19.6 TABLE S4 Rhinoceros species and phytoestrogen profiles significantly interact to affect estrogen receptor activation. Download Table S4, DOCX file, 0.01 MB. Copyright © 2019 Williams et al. 2019 Williams et al. This content is distributed under the terms of the Creative Commons Attribution 4.0 International license.

### Interactions with SWR fertility explored.

To assess the fertility of our SWR population, the number of pregnancies achieved and/or calves born was determined for both the period of sample collection as well as for the lifetime of each of the SWR included in this study (Table S1). Pregnancies achieved (Pregnancy_study_ [PS] or Pregnancy_life_ [PL]) were confirmed via elevations in fecal progestagen levels and were included in the analysis since rhino gestation length (∼16 months) exceeded the duration of sample collection (4 months). Fertility (Calf_study_ [CS] or Calf_life_ [CL]) represents calves born per reproductive year using calculations described previously (4). When comparing phytoestrogen profiles using CS, we did not find any significant difference in mean fertility (Fig. 4G to I; see Table S5 in the supplemental material). Using the PS calculation, however, we showed that individuals exhibiting profile A had the lowest mean pregnancy rate, and those producing profile C had the highest (Fig. 4G and I). For lifetime measures, we found a similar relationship, with PL and CL for profile C producers being significantly greater than those for profile A producers (Fig. 4G and I). Although not significantly different, SWR producing profile B profiles tended to have higher mean fertility than individuals belonging to profile A across all measures (Fig. 4G and H).

10.1128/mBio.00311-19.7 TABLE S5 Significant interactions between SWR fertility and phytoestrogen profiles, including correlations between fertility and individual fecal analytes. Download Table S5, DOCX file, 0.1 MB. Copyright © 2019 Williams et al. 2019 Williams et al. This content is distributed under the terms of the Creative Commons Attribution 4.0 International license.

Although not all were significant, 12 interactions with fertility measurements were observed across six OTUs (see Table S6 in the supplemental material). Two of these OTUs, a member of the RC9 gut group (OTU 46) and an unclassified *Lachnospiraceae* (OTU 97), were significantly correlated to increased fertility. The remaining four OTUs associated with decreased fertility were comprised of members from the *Bacteroidales* (OTU 34), YRC22 group (OTU 42), RC9 gut group (OTU 92), and *Prevotella* spp. (OTU 193). We found that the combined relative abundance of positively associated OTUs contributed to 52% of the variation observed in CS (Table S6), but the abundance of negatively associated OTUs did not, with percentages of variation observed ranging from 1.6% to 10% across the four measures (Table S6). However, only OTUs negatively correlated to fertility displayed significant interactions with any microbial metabolite examined in our study, with three of the four OTUs (OTUs 34, 42, and 193) displaying significant negative correlations to both microbial metabolites EQ and EL.

10.1128/mBio.00311-19.8 TABLE S6 Interactions between SWR fertility and microbiota. Download Table S6, DOCX file, 0.1 MB. Copyright © 2019 Williams et al. 2019 Williams et al. This content is distributed under the terms of the Creative Commons Attribution 4.0 International license.

## DISCUSSION

Working with threatened species, such as the two rhinoceros species studied here, presents its own unique set of challenges. Despite these challenges, however, our combining of parallel sequencing, mass spectrometry, and estrogen receptor activation assays provides insight into the host-microbe relationship with fertility that, to our knowledge, is novel for any vertebrate species. Such an approach is needed to understand and apply novel application of techniques within nontraditional systems.

Although microbial communities differed between SWR and GOHR, the overall phytoestrogen metabolites each species produce were similar. The observed differences in microbial community are likely related to the different foraging strategies exhibited by the two species. All individuals in this study live in large exhibits where they are provided diet of soy- and alfalfa-based pellets and supplemented with either grasses or browse. SWR, which in the wild are grazers, consume additional hay and fresh grasses (8, 9). In contrast, GOHR, a predominantly browsing species, consume a more varied diet that includes fruits and leaves (27). This difference in foraging may be driving species differences in gut microbiota, as observed in other closely related species (28). However, there may be other factors at play driving differences in gut microbiota, such as age differences between individuals (29) and their previous medical history, such as previous antibiotic use (30). Nevertheless, both species are herbivorous, and their gut microbiotas are similar in that the dominant microorganisms present in both species are related to those capable of fiber degradation (31). The likelihood that these dominant microbiotas fulfill similar functional niches is one possible explanation for similarity in phytoestrogen compositions between SWR and GOHR. That is, OTUs positively associated with phytoestrogen concentrations are closely related to taxa that are known fiber degraders. Although our representative OTUs are poorly classified in many cases, it is possible that members of the *Bacteroidetes* (*Rikenellaceae* and *Prevotellaceae*) in SWR and the *Firmicutes* (*Ruminococcaceae* and *Lachnospiraceae*) in GOHR may contribute to metabolite production in addition to fiber degradation via β-glucosidase activity, as this enzyme also catalyzes early steps of phytoestrogen transformation (23). Thus, the lack of species differences in phytoestrogen composition may be driven by the overall functional similarity of the two species’ gut microbial communities.

With some exceptions, estrogenicity of the three dominant fecal phytoestrogen metabolite profiles identified by hierarchical clustering followed expected patterns. The profile observed with the highest overall level of phytoestrogens displayed the highest levels of activation across both ERs, yet profile B stimulated similar levels of activation of ERs from both rhino species, despite containing half the total metabolites of profile C. We attribute this high activation by both profiles primarily to EQ, a dominant metabolite in both profiles and a known potent agonist to rhino ERs (4). However, it is interesting that activation of SWR ERα by profiles B and C was significantly greater than that of GOHR ERα (Table S4), as previous work has shown GOHR ERα to be slightly more sensitive to EQ than its SWR homologue (4). Interestingly, profile A was a more potent agonist of GOHR ERβ compared to SWR ERβ. This is noteworthy, as no single phytoestrogen tested in previous studies has ever been shown to be a more potent agonist of GOHR ERβ than SWR ERβ. What is driving these differences is unclear, as the dominant metabolites in profile A, EL and PEP, do not appreciably bind or activate ERβs from either species, and the known agonists present in profile A (DZ, EQ, and CO) are more potent activators of SWR ERβs than those from GOHR (4). Nevertheless, this observation highlights the importance of evaluating the effects of mixtures of suspected endocrine-disrupting chemicals on receptors, in addition to individual chemicals, as this method better mimics *in vivo* conditions.

Mean reproductive success, in terms of pregnancies achieved and calves born, was highest in individuals with the greatest concentrations of fecal metabolites (Fig. 4I; profile C). For some of the metabolites produced, these findings parallel observations by others. For example, all measures of SWR fertility were positively correlated with production of EL (Table S5). This finding is consistent with studies in humans that have demonstrated a link between high levels of EL and increased reproductive success (32). Our previous work shows EL does not appreciably bind or activate SWR ERs and therefore possesses little endocrine-disrupting potential as a xenoestrogen (4). However, the positive relationship between EQ and calf-based fertility measures is unexpected (Table S5). *In vitro*, EQ is a relatively potent agonist of both SWR ERα and ERβ (4), and in other vertebrate species, EQ is cleared from the circulation less quickly than other isoflavones, increasing its bioavailability (33). This suggests that high levels of EQ production should negatively affect SWR fertility, as is well documented in other grazing species (11, 12).

Another unexpected finding was that individual SWR producing the most estrogenic profiles (profiles B and C) exhibited the highest fertility (Fig. 4H and I), while SWR fertility was lowest in individuals producing profiles with the lowest overall estrogenicity (profile A). These observations lead to several new questions. Do SWR belonging to profile A possibly produce novel phytoestrogen metabolites that are more estrogenic that were not detected by our targeted approach? We observed high levels of certain compounds, such as MOC and CO in feeds, but low levels were detected in feces. CO is a potent SWR ER agonist (4) and has been associated with infertility in sheep (11), but little is known about possible microbial metabolites and their relative estrogenicity. It is possible that these coumestans are converted into a novel metabolite that could be highly estrogenic to SWR. Another possible explanation for the positive association between profile estrogenicity and fertility is that the various degrees of fecal profile estrogenicity result from differences in phytoestrogen absorption or excretion between individuals. Specifically, it could be hypothesized that elevated excretion of phytoestrogens and metabolites would reduce circulating levels, thus, limiting the potential for these chemicals to cause reproductive harm. This is supported not only by our findings in individual animals, but also by our species-level observations where the more fertile GOHR generally excrete higher levels of phytoestrogens than the less fertile SWR. This does not appear to be case in in sheep and cattle, where concentrations of phytoestrogens and metabolites in excreta (i.e., urine) generally correlate to plasma levels (34–36). However, detailed studies examining the generation and clearance of phytoestrogen metabolites, as well as their subsequent endocrine-disrupting effects on target tissues, are lacking even for relatively well-studied species. Addressing such relationships in SWR will be challenging, if not impossible. Nevertheless, the findings presented here do provide the opportunity to apply potentially innovative approaches, like using nontargeted mass spectrometry to identify novel metabolites or using fecal EL or EQ concentrations to identify individual SWR with high reproductive potential.

Few studies have examined the interaction between mammalian fertility and gut microbiota (37), and defining this link is difficult. Here, we found the abundance of six OTUs to correlate to fertility measures (Table S6). None of these taxa have been previously associated with fertility status in other mammalian species, and it is unknown what role these microbiota may play. Using correlations between microbial abundance and phytoestrogen metabolites to determine microbial activity is biased, as compositional data, like those presented here, do not directly correlate to microbial activity (38): for example, members of the *Coriobacteriaceae* (*Slackia*, *Eggerthella*, and *Adlercreutzia*), which have been shown to transform DZ to EQ (39), the *Eubacteriaceae* (*Eubacterium*), which are capable of dehydroxylation of lignans to produce ED and EL (40), and the *Blautia* spp., which have displayed both lignan and isoflavone metabolism (20, 39) and are found in samples collected from both SWR and GOHR in low abundances (all at <1.0%). Despite the low abundance of known phytoestrogen metabolizers, both rhinoceros species excrete large amounts of phytoestrogen metabolites, making it likely that less abundant taxa may significantly contribute to the transformation of phytoestrogens. However, further work is needed to determine which microbiota are contributing to phytoestrogen metabolism within the rhinoceros, including *in vitro* culture experiments to measure their microbial activity.

Finally, our work sheds light on how microbiota may drive reproductive outcomes in SWR, but they are not the only species that may benefit from the work presented here. To our knowledge, no previous work has combined the above approaches to examine how microbially mediated phytoestrogen metabolism may relate to fertility in any vertebrate species, and this study may serve as the first to better inform us of the role microbiota may play in endocrine disruption and negative host outcomes. Among its broader application to other vertebrates, however, our findings may be critical for the management of SWR’s closest relative, the northern white rhinoceros (NWR [*Ceratotherium simum cottoni*]), a subspecies with only two living members (41). Like SWR, NWR experience low fertility and a prevalence of reproductive pathologies in managed settings (17). As a closely related grazing subspecies, the NWR is likely sensitive to phytoestrogens as well. Currently, several rescue attempts are under way to prevent NWR extinction (42, 43). Should these attempts to save the NWR be successful, and with SWR facing a similar uncertain fate, any novel approaches to promote high fertility, such as managing microbial phytoestrogen transformation by altering microbiota through diet modifications and other therapeutic approaches will be needed. With the information presented here, we plan to direct future work aimed at developing strategies to improve captive SWR reproduction, with the ultimate goal of alleviating their threat of extinction.

## MATERIALS AND METHODS

### Study animals.

The female greater one-horned rhinoceros (*n = *2) and southern white rhinoceros (*n = *6) used in this study were housed at the San Diego Zoo Safari Park, Escondido, CA, in two separate 24-ha mixed-species exhibits (Table S1). All procedures were approved by San Diego Zoo Global’s Institutional Animal Care and Use Committee (no. 15-013).

### Sample collection.

Fresh fecal samples (SWR, *n = *42; GOHR, *n = *16) (Table S1) were collected weekly beginning 3 September 2015 through 1 January 2016, alternating weeks between SWR and GOHR. Samples were collected from animals at the same time of day using binoculars to identify individuals based on their unique horn structure. Following defecation, collection occurred between 1 to 20 min, and samples were transported on dry ice and stored at −80°C prior to processing.

### DNA extraction.

Total genomic DNA from fecal samples and the negative control was extracted via mechanical disruption and hot/cold phenol extraction following Stevenson et al.’s protocol (44), with the exception that 25:24:1 phenol-chloroform-isoamyl alcohol was used in place of phenol-chloroform at all steps. DNA was quantified using a Qubit fluorometer (Invitrogen, Carlsbad, CA) and stored at −20°C following extraction.

### Library preparation and sequencing.

Sequencing library preparation was carried out following the manufacturer’s recommendations (Illumina, 2013) with some modifications. In brief, amplicon PCR targeted the V4 region of the 16S rRNA gene using a forward primer (V4f: TATGGTAATTGTGTGCCAGCMGCCGCGGTAA) and reverse primer (V4r: AGTCAGTCAGCCGGACTACHVGGGTWTCTAAT) in a 25-μl reaction mixture with 1× KAPA HiFi Hot Start Ready mix (Kapa Biosystems), 0.2 mM each primer, and 1.0 to 5.0 ng DNA (32). Amplification conditions were as follows: 95°C for 2 min, followed by 25 cycles of 95°C for 20 s, 55°C for 15 s, and 72°C for 30 s and a final 10-min extension at 72°C. PCR products were purified via gel extraction (Zymo Gel DNA recovery kit; Zymo, Irvine, CA) using a 1.0% low-melt agarose gel (National Diagnostics, Atlanta, GA) and quantified with a Qubit fluorometer (Invitrogen). With the negative control producing no band, the expected area was excised. All samples were combined to yield an equimolar 4 nM pool. Following the manufacturer’s protocol, sequencing was conducted on an Illumina MiSeq using reagent kit V2 (2× 250-bp cycles), as described previously (Illumina, 2013).

### 16S rRNA sequence analyses.

Sequence analysis was carried out using mothur v.1.39.5 (45) following the MiSeq standard operating procedure (SOP) (46). In brief, contigs were formed from 16S rRNA reads, and poor-quality sequences were removed. Sequences were trimmed and filtered based on quality (maxambig = 0, minlength = 250, maxlength = 500). Unique sequences were aligned against the SILVA 16S rRNA gene alignment database (47) and classified with a bootstrap value cutoff of 80, and operational taxonomic units (OTUs) found with <2 sequences in the total data set were removed. Chimeras (chimera.uchime), sequences identified as members of the *Eukaryota*, *Archaea*, and *Cyanobacteria* lineages, and mitochondria were also removed. Sequences were clustered into OTUs at a 97% similarity cutoff using OptiClust (see Table S7 in the supplemental material). The negative control yielded 273 sequences, comprised of low-level cross-sample contaminants; therefore, OTUs were not removed from the data set.

Sequence coverage was assessed in mothur by rarefaction curves (see Fig. S2 in the supplemental material) and Good’s coverage (48). Samples were then iteratively subsampled 10 times to 6,825 sequences per sample, and OTU abundances were calculated as whole number means across iterations. Additionally, richness and diversity were calculated for each sample. All other calculations were carried out in R using both *vegan* and *phyloseq* packages (49, 50). The similarity indices Bray-Curtis (51), Jaccard (52), and weighted UniFrac (53) were used to assess differences in bacterial community, and these differences were visualized by nonmetric multidimensional scaling (nMDS [iters = 10,000]) plots (54). Permutational analysis of multivariate dispersions (PERMDISP2) was used to test for heterogeneity of community structure and composition between rhino species, and with unequal variances observed, data were down-sampled to create even sample sizes using the *caret* package (55) prior to permutational analysis of variance (PERMANOVA [*vegan*::adonis; SWR, *n = *16; GOHR, *n = *16]) to determine species differences. Similarity percentages (SIMPER [*vegan*]) analyses then determined the contributions from each taxonomic group to PERMANOVA reported differences. Species-related differences in individual OTUs were examined by Welch’s *t* test (two-sided, SWR, *n = *16; GOHR, *n = *16). All data are expressed as the mean ± standard error (SE) and considered significant if *P* is <0.05 unless otherwise stated.

10.1128/mBio.00311-19.2 FIG S2 Rarefaction results based on 97% operational taxonomic units (OTUs) for all greater one-horned rhinoceros (GOHR) and southern white rhinoceros (SWR) samples. Download FIG S2, TIF file, 0.5 MB. Copyright © 2019 Williams et al. 2019 Williams et al. This content is distributed under the terms of the Creative Commons Attribution 4.0 International license.

### Phytoestrogen extraction and quantification.

Samples collected were batched into groups of 10 and accompanied by quality control samples. Phytoestrogens were extracted from fecal samples by a two-phase extraction as described previously by Palme et al. (56) with few modifications. In the first phase, fecal samples were diluted 10-fold using 80% methanol in water (Fisher Scientific), homogenized for 20 min using a Geno/Grinder at 1,000 rpm, and centrifuged for 10 min at 4,000 × *g*, and the supernatant was recovered. In the second phase, 1.0 ml of methanol extract was added to 4.0 ml diethyl ether (Fisher Scientific), 0.5 ml of 5.0% NaH_2_CO_3_ (Sigma), and 4.0 ml of water (57), inverted four times, and centrifuged for 10 min at 4,000 × *g*. The ether phase was removed, evaporated at 45°C by a nitrogen flow of 0.4 lb/in^2^, and resuspended in methanol. Extracts were further filtered (0.22 μm) and analyzed by liquid chromatography-coupled tandem mass spectrometry (LC-MS/MS) for all analytes, with the exception of 4′-ethylphenol (PEP), which was analyzed by a gas chromatography mass selective detector (GC-MSD). Quality control samples included a blank matrix sample (grass) that was absent of phytoestrogens to assess contamination during the extraction and a spiked-matrix sample that was fortified with a known concentration of phytoestrogens. The spiked-matrix sample was used to determine the efficiency of the extraction for every batch: recoveries ranged between 50 and 150%.

### LC-MS/MS method.

Analysis was performed using an Agilent 1260 liquid chromatograph coupled to an Agilent 6430 triple mass spectrometer. Chromatographic separation was performed using an Agilent Zorbax Eclipse Plus (2.1 by 50-mm inside diameter [i.d.], 1.8 µm) Rapid Resolution column maintained at 40°C. The mobile phases consisted of 5 mM ammonium formate and 0.1% formic acid in water for the aqueous phase (A), with 5 mM ammonium formate and 0.1% formic acid in methanol as the organic phase (B). The flow rate was held at 0.4 ml/min, and the gradient program was as follows: 0 to 0.5 min of 10% B followed by 0.5 to 3.0 min of increase to 90% B. The ionization of phytoestrogens was performed using electrospray ionization (ESI) in positive mode with an auxiliary gas (N_2_), source temperature of 300°C, and a gas flow rate of 12 liters/min, with the exception of enterodiol, which was run in negative mode. Optimized multiple-reaction-monitoring (MRM) conditions are listed in Table S8.

10.1128/mBio.00311-19.10 TABLE S8 Phytoestrogen standards and their optimized MRM conditions for LC-MS/MS. Download Table S8, DOCX file, 0.1 MB. Copyright © 2019 Williams et al. 2019 Williams et al. This content is distributed under the terms of the Creative Commons Attribution 4.0 International license.

### GC-MSD method.

The analysis of PEP (Indofine, CAS: 123-07-09) was performed on an Agilent 7890B gas chromatograph (GC) coupled to an Agilent 5977 A mass selective detector (MSD). The GC inlet temperature was set to 280°C run in pulsed splitless mode with an injection volume of 1 μl. The GC oven temperature was set to 80°C and increased to 200°C between 1 and 13 min at a rate of 10°C/min. The oven temperature was then increased to 300°C between 13 and 22 min at a rate of 25°C/min for a total run time of 22 min. Ultrahigh-purity helium (carrier gas) was used at a constant flow rate of 1.5 ml/min with an Agilent DB-5MS UI (30 m by 0.250 mm) 0.25-μm analytical column. PEP was analyzed using an electron ionization (EI) source with a source temperature of 230°C. The selected ion monitoring (SIM) mode was to monitor 77, 107, and 122 (*m*/*z*) ions with a gain factor of 10 and a scan speed of 1,562 (u/s).

### Phytoestrogen analyses.

Similar methods to those used for 16S rRNA analyses were used in determining species differences in phytoestrogen analyte composition. Differences were visualized following nMDS of Bray-Curtis and Jaccard similarity indices, and following normalization, normality testing, and down-sampling, PERMANOVA was used to determine if species differences were observed (SWR, *n = *16; GOHR, *n = *16). Welch’s *t* test was again used to measure significant differences between rhino species for individual analytes (SWR, *n = *16; GOHR, *n = *16). Since we did not observe a species-related difference using PERMANOVA, and no apparent clustering was observed with nMDS, hierarchical clustering (Bray-Curtis) was used to group phytoestrogen data into three profiles (A, B, and C) based on their compositional similarity for further analyses. SIMPER analysis was used to determine contributions of each analyte to differences observed, and significant differences between groups were tested using analysis of variance (ANOVA; profile A, *n *=* *23 [SWR, 11; GOHR, 1]; profile B, *n *=* *26 [SWR, 15; GOHR, 11]; profile C, *n =* 9 [SWR, 4; GOHR, 4]) with FDR correction.

### Receptor activation.

The ability of phytoestrogens and metabolites to activate SWR ERs was assessed using an SWR and GOHR estrogen receptor (ER) activation assay described previously by Tubbs et al. (4, 7) with minor modification. For each species, ERα or ERβ subcloned into pcDNA3.1(+) expression plasmid (Invitrogen) was cotransfected into human embryonic kidney (HEK293) cells along with pCMX-β-galactosidase (β-Gal) and pGL2–3xERE luciferase reporter plasmids. After 24 h, cells were treated with phytoestrogens or metabolites, alone or in combination, and incubated for an additional 24 h. For single test compounds, cells were treated with 100 pM to 10 μM each compound or vehicle (dimethyl sulfoxide [DMSO]) alone. To assess the estrogenicity of phytoestrogen/metabolite profiles produced by SWR and GOHR microbial communities, cells were treated with serial dilutions of mixtures created *in vitro* to reflect those generated *in vivo*. Within each assay, a series of cells was treated with the endogenous estrogen, 17-estradiol (E_2_ [0.001 to 100 nM]), to determine maximal E_2_ activation. Following incubation, cells were lysed, and luciferase and β-Gal activities were measured as described previously (7). All data are presented as mean ± SE fold activation over vehicle treatment for each metabolite or mixture relative to the maximal activation of E_2_. Differences in mean activation for both ERs were determined by ANOVA with FDR correction (each treatment, *n = *9 [interassay, *n *=* *3; intra-assay, *n *=* *3]), and significant interactions were observed between rhinoceros species and phytoestrogen profile. Data were sliced according the main effects, and differences within factors were observed.

### Fertility.

Four calculations of SWR fertility were conducted for this study. Similar to Tubbs et al. (4), two calculations are based on the number of offspring produced by the female per reproductive year through the completion of the study (Calf_study_ [CS]) and current levels (Calf_life_ [CL]). With pregnancies also considered a success, we conducted two additional calculations based on the number of pregnancies per reproductive year ending with the completion of the study (Pregnancy_study_ [PS]) through current (Pregnancy_life_ [PL]). GOHR samples were removed from the data set so that data were not skewed by species differences.

### Correlations.

Using the *microbiome* package in R (58), we examined significant correlations between OTUs (≥1.0% relative abundance) and phytoestrogen analytes, OTUs and fertility measures, and phytoestrogen analytes and fertility measures. These correlations were carried out using the Spearman correlation method with multiple-testing correction by FDR (p.adjust, *stats* package, *P* < 0.05). OTUs found to correlate to fertility were further examined using a linear model (lm, *stats* package) (59).

### Data availability.

All sequences have been deposited into the National Center for Biotechnological Information’s Short Read Archive under SRA accession no. SRP136468.
