# Supplementary material for: Gut Microbiota and Phytoestrogen-Associated Infertility in Southern White Rhinoceros
Source: mBio. 2019 Apr 9;10(2):e00311-19. doi: 10.1128/mBio.00311-19 (PMC6456749; doi:10.1128/mBio.00311-19)
Supplement: TABLE S1 [file mBio.00311-19-st001.docx]

**Table S1**. Study Animals

| **Studbook**  **No.** | **Accession No.** | **Species** | **DOB** | **Samples (n)** | **Fertility** | | | |
| --- | --- | --- | --- | --- | --- | --- | --- | --- |
|  |  |  |  |  | **CS** | **PS** | **CL** | **PL** |
| 819 | 026210 | SWR | 24 June 1984 | 8 | 0 | 0 | 0 | 0 |
| 1237 | 600278 | SWR | 10 Dec 1998 | 7 | 0 | 0.15 | 0.067 | 0.20 |
| 1394 | 606103 | SWR | 16 Nov 2001 | 7 | 0 | 0.10 | 0.083 | 0.17 |
| 1594 | 609372 | SWR | 16 Aug 2008 | 7 | 0 | 0 | 0 | 0 |
| 1613 | 609373 | SWR | 20 Feb 2007 | 6 | 0.50 | 0.50 | 0.50 | 0.50 |
| 1151 | 697039 | SWR | 30 Jan 1997 | 7 | 0 | 0 | 0 | 0 |
| 349 | 608199 | GOHR | 26 Dec 2004 | 8 | 0.43 | 0.43 | 0.33 | 0.44 |
| 372 | 606309 | GOHR | 29 Sept 2006 | 8 | 0.60 | 0.60 | 0.43 | 0.57 |

SWR: southern white rhinoceros; GOHR: greater one-horned rhinoceros; DOB: date of birth; CS: calf-based/study period; PS: pregnancy-based/study period; CL: calf-based/lifetime; PL: pregnancy-based/lifetime.
