# Supplementary material for: Gut Microbiota and Phytoestrogen-Associated Infertility in Southern White Rhinoceros
Source: mBio. 2019 Apr 9;10(2):e00311-19. doi: 10.1128/mBio.00311-19 (PMC6456749; doi:10.1128/mBio.00311-19)
Supplement: TABLE S2 [file mBio.00311-19-st002.docx]

**Table S2**. Number of sequences, estimate coverage, diversity and OTU richness of samples.

| Samples (*n*) | High quality sequences | Richness | | | | Diversity |
| --- | --- | --- | --- | --- | --- | --- |
|  |  | Inverse Berger Parker | Shannon | Inverse-Simpson | Unique OTUs | Good’s coverage |
|  |  | *P* = 0.017* | *P* = 0.015* | *P* = 0.015* |  |  |
| SWR (*n =* 42) | 28,062 ± 2080 | 6.9 ± 0.31 | 4.2 ± 0.054 | 22 ±1.3 | 766 | > 0.98 |
| GOHR (*n =* 16) | 24,092 ± 1952 | 9.6 ± 0.88 | 4.6 ± 0.10 | 37 ±4.6 | 433 | > 0.98 |

Mean ± SEM given.*Significance tested (*t*-test, *P* < 0.05). All p-values are adjusted by FDR. SWR: southern white rhinoceros; GOHR: greater one-horned rhinoceros.
