# Supplementary material for: Gut Microbiota and Phytoestrogen-Associated Infertility in Southern White Rhinoceros
Source: mBio. 2019 Apr 9;10(2):e00311-19. doi: 10.1128/mBio.00311-19 (PMC6456749; doi:10.1128/mBio.00311-19)
Supplement: TABLE S3 [file mBio.00311-19-st003.docx]

**Table S3**. Significant contributions to variation to species differences and phytoestrogen profiles by individual analytes.

| **Analyte** | **Species** | **Phytoestrogen Profiles** | | | | | | |
| --- | --- | --- | --- | --- | --- | --- | --- | --- |
|  | **Adj. P** | **Simper (%)** | | | **Adj. P-value** | | | |
|  |  | **A:B** | **A:C** | **B:C** | **Overall** | **A:C** | **A:B** | **B:C** |
| EQ | 0.031* | 42 | 66 | 59 | <0.001* | <0.001* | <0.001* | <0.001* |
| EL | 0.032* | 29 | 19 | 19 | 0.0017* | 0.0033* | 0.018* | >0.05 |
| PEP | >0.05 | 19 | 10 | 13 | >0.05 | -- | -- | -- |
| CO | <0.001* | 5.7 | 1.9 | 2.7 | <0.001* | 0.0018* | <0.001* | >0.05 |
| ED | >0.05 | 1.1 | 1.8 | 2.2 | <0.001* | <0.001* | >0.05 | 0.0040* |
| DZ | >0.05 | 3.0 | 1.2 | 1.4 | >0.05 | -- | -- | -- |
| MOC | 0.0042* | 0.64 | 1.1 | 1.6 | >0.05 | -- | -- | -- |

*Significance tested (*t*-test/ANOVA, *P* < 0.05). All p-values are adjusted by FDR. EQ: equol; EL: enterolactone, PEP: 4’ethylphenol, CO: coumestrol, ED: enterodiol, DZ: daidzein, MOC: methoxycoumestrol.
