# Supplementary material for: Gut Microbiota and Phytoestrogen-Associated Infertility in Southern White Rhinoceros
Source: mBio. 2019 Apr 9;10(2):e00311-19. doi: 10.1128/mBio.00311-19 (PMC6456749; doi:10.1128/mBio.00311-19)
Supplement: TABLE S4 [file mBio.00311-19-st004.docx]

**Table S4**. Rhinoceros species and phytoestrogen profiles significantly interact to affect estrogen receptor activation.

| **Comparison** | | **ERα** | **ERβ** |
| --- | --- | --- | --- |
| Species*Profile | | <0.0001* | 0.010* |
| SWR | Overall | <0.0001* | <0.0001* |
|  | A:B | <0.0001* | <0.0001* |
|  | A:C | <0.0001* | <0.0001* |
|  | B:C | 0.0053* | 0.001* |
| GOHR | Overall | <0.0001* | <0.0001* |
|  | A:B | <0.0001* | <0.0001 |
|  | A:C | <0.0001* | <0.0001* |
|  | B:C | 0.058 | 0.0013* |
| A | SWR:GOHR | 0.53 | 0.0008* |
| B | SWR:GOHR | 0.00028* | 0.82 |
| C | SWR:GOHR | <0.0001* | 0.12 |

*Significance tested (ANOVA, *P* < 0.05, FDR correction).
