# Supplementary material for: Gut Microbiota and Phytoestrogen-Associated Infertility in Southern White Rhinoceros
Source: mBio. 2019 Apr 9;10(2):e00311-19. doi: 10.1128/mBio.00311-19 (PMC6456749; doi:10.1128/mBio.00311-19)
Supplement: TABLE S5 [file mBio.00311-19-st005.docx]

**Table S5**. Significant interactions between SWR fertility and phytoestrogen profiles, including correlations between fertility and individual fecal analytes.

| **Fertility measure** |  | **Profile (Adj. P-value)** | | | **Spearman’s ρ** | | | | | |
| --- | --- | --- | --- | --- | --- | --- | --- | --- | --- | --- |
|  | **Overall** | **A:B** | **A:C** | **B:C** | **DZ** | **EQ** | **PEP** | **ED** | **EL** | **CO** |
| CS | 0.094 | -- | -- | -- | -0.30 | +0.37* | +0.22 | +0.11 | +0.39* | +0.47* |
| CL | 0.043* | >0.05 | 0.028* | >0.05 | -0.026 | +0.36* | +0.25 | +0.32 | +0.63* | +0.23 |
| PS | 0.039* | >0.05 | 0.035* | >0.05 | -0.048 | +0.33 | +0.36* | +0.35* | +0.62* | +0.25 |
| PL | 0.025* | >0.05 | 0.021* | >0.05 | -0.048 | +0.33 | +0.36* | +0.35* | +0.62* | +0.25 |

*Significance tested (ANOVA; *P* < 0.05). All p-values are adjusted by FDR. CS: calf-based/study period; PS: pregnancy-based/study period; CL: calf-based/lifetime; PL: pregnancy-based/lifetime.
