# Supplementary material for: Gut Microbiota and Phytoestrogen-Associated Infertility in Southern White Rhinoceros
Source: mBio. 2019 Apr 9;10(2):e00311-19. doi: 10.1128/mBio.00311-19 (PMC6456749; doi:10.1128/mBio.00311-19)
Supplement: TABLE S6 [file mBio.00311-19-st006.docx]

**Table S6.** Interactions between SWR fertility and microbiota.

| **OTU** | **CS** | | | **CL** | | | **PS** | | | **PL** | | |
| --- | --- | --- | --- | --- | --- | --- | --- | --- | --- | --- | --- | --- |
|  | **ρ** | **Adj. P** | **R^2^** | **ρ** | **Adj. P** | **R^2^** | **ρ** | **Adj. P** | **R^2^** | **ρ** | **Adj. P** | **R^2^** |
| **Positive** | -- | <0.001* | 0.52 | -- | <0.001* | 0.44 | -- | <0.001* | 0.36 | -- | <0.001* | 0.27 |
| OTU 46 | +0.48 | <0.001* | 0.42 | +0.12 | <0.001* | 0.36 | +0.11 | <0.001* | 0.29 | +0.11 | <0.001* | 0.22 |
| OTU 97 | +0.46 | <0.001* | 0.35 | +0.18 | <0.001* | 0.30 | +0.16 | <0.001* | 0.25 | +0.16 | 0.0023* | 0.19 |
| **Negative** | -- | >0.050 | 0.016 | -- | >0.050 | 0.048 | -- | >0.050 | 0.084 | -- | >0.050 | 0.10 |
| OTU 42 | -0.53 | >0.050 | -- | -0.53 | >0.050 | -- | -0.46 | >0.050 | -- | -0.24 | 0.022* | 0.10 |
| OTU 34 | -0.47 | >0.050 | -- | -0.47 | >0.050 | -- | -0.43 | >0.050 | -- | -0.36 | >0.050 | -- |
| OTU 92 | -0.47 | >0.050 | -- | -0.47 | >0.050 | -- | -0.44 | >0.050 | -- | -0.23 | >0.050 | -- |
| OTU 193 | -0.47 | >0.050 | -- | -0.48 | >0.050 | -- | -0.48 | >0.050 | -- | -0.30 | >0.050 | -- |

*Significance tested (*P* < 0.05) using linear model, and all p-values are adjusted by FDR, and Spearman’s **ρ** provided. CS: calf-based/study period; PS: pregnancy-based/study period; CL: calf-based/lifetime; PL: pregnancy-based/lifetime. OTU 46: RC9 group; OTU 97: Lachnospiraceae; OTU 42: Bacteroidales; OTU 34: Bacteroidales; OTU 92: RC9 group; OTU 193: *Prevotella* spp.
