# Supplementary material for: Gut Microbiota and Phytoestrogen-Associated Infertility in Southern White Rhinoceros
Source: mBio. 2019 Apr 9;10(2):e00311-19. doi: 10.1128/mBio.00311-19 (PMC6456749; doi:10.1128/mBio.00311-19)
Supplement: TABLE S8 [file mBio.00311-19-st008.docx]

**Table S8.** Phytoestrogen standards and their optimized MRM conditions for LC/MS/MS.

| **Phytoestrogen** | **CAS** | **Source** | **LC/MS/MS MRM condition** | | | | |
| --- | --- | --- | --- | --- | --- | --- | --- |
|  |  |  | **Precursor Ion (m/z)** | **Product Ion (m/z)** | **Fragmentor** | **Collision Energy (eV)** | **Polarity** |
| 4' Methylcoumestrol | 1690-62-6 | Quality Pharmaceuticals | 283.1 | 255.1 | 172 | 20 | + |
|  |  |  |  | 227.1 |  | 25 |  |
| Biochanin-A | 491-80-5 | Sigma | 285.1 | 213 | 133 | 41 | + |
|  |  |  |  | 152 |  | 29 |  |
| Coumestrol | 479-13-0 | Indofine | 269.1 | 213 | 123 | 29 | + |
|  |  |  |  | 157 |  | 37 |  |
| Daidzein | 486-66-8 | Indofine | 255.1 | 199.1 | 148 | 25 | + |
|  |  |  |  | 91.1 |  | 45 |  |
| Enterolactone | 78743-71-9 | Sigma | 299.1 | 107 | 133 | 29 | + |
|  |  |  |  | 133 |  | 13 |  |
| Equol | 531-95-3 | Indofine | 243.1 | 133 | 79 | 17 | + |
|  |  |  |  | 123 |  | 9 |  |
| Fomononetin | 485-72-3 | Sigma | 269.1 | 253 | 143 | 29 | + |
|  |  |  |  | 237 |  | 25 |  |
| Genistein | 446-72-0 | Indofine | 271.1 | 153 | 143 | 29 | + |
|  |  |  |  | 91.1 |  | 45 |  |
| *o*-desmethylangolesin | 21255-69-6 | Sigma | 259.1 | 149 | 99 | 9 | + |
|  |  |  |  | 121 |  | 25 |  |
| Enterodiol | 80226-00-2 | Sigma | 301.1 | 253.1 | 133 | 16 | - |
|  |  |  |  | 106 |  | 32 |  |
